# Supplementary figures and images for: Viruses in saliva from sanctuary chimpanzees (Pan troglodytes) in Republic of Congo and Uganda
Source: PLoS One. 2023 Jun 29;18(6):e0288007. doi: 10.1371/journal.pone.0288007 (PMC10310015; doi:10.1371/journal.pone.0288007)

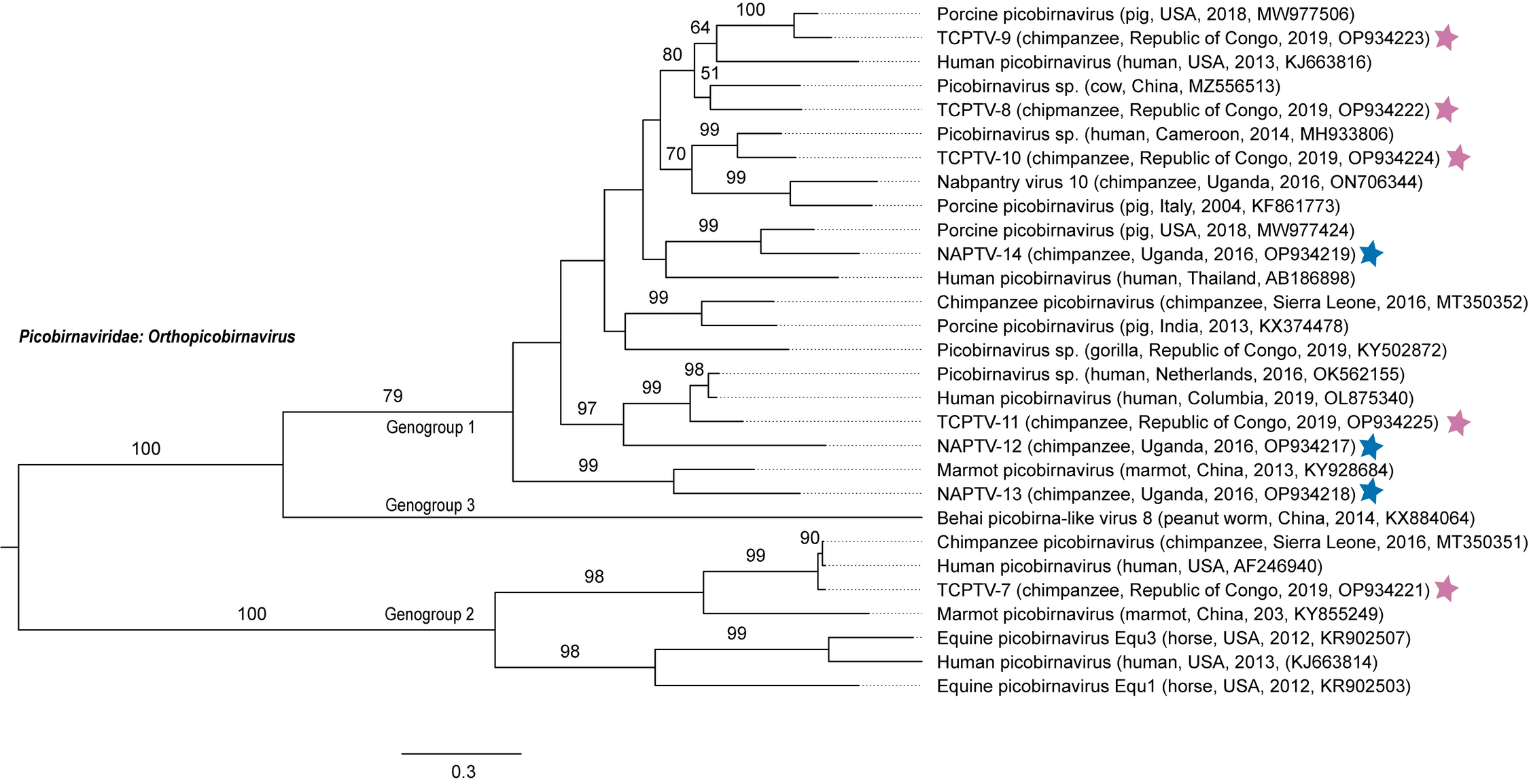

Supplement: S1 Fig — Viruses identified in this study are marked with a colored star to indicate the sanctuary of origin (purple = TCRC, blue = NICS). Virus names are followed by (host, location, year, GenBank accession number). Bootstrap values (%) ≥ 50 are represented by numbers beside branches (1000 replicates). Scale bar is equal to nucleotide substitutions per site. (TIF) [file pone.0288007.s001.tif]

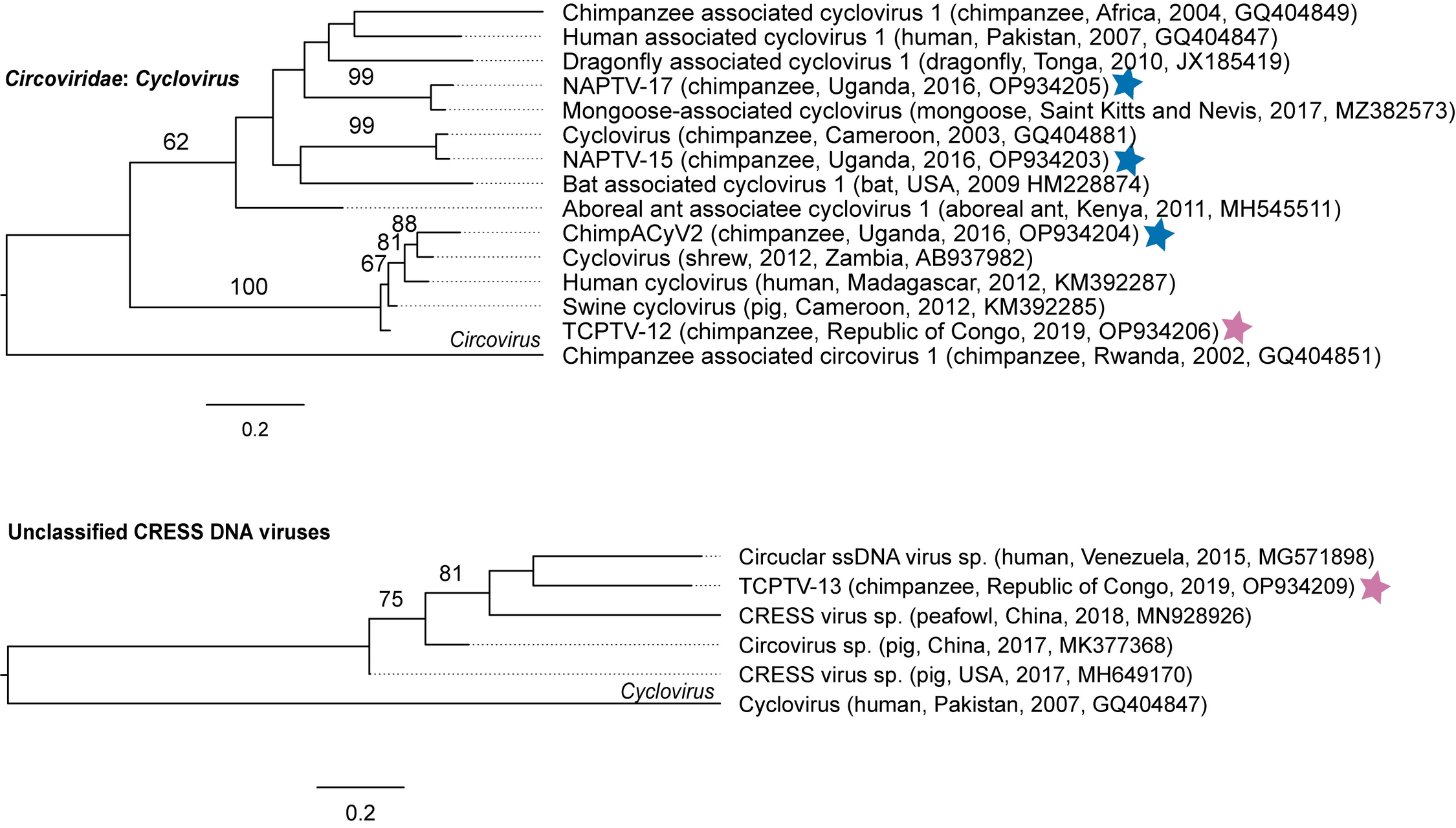

Supplement: S2 Fig — Viruses identified in this study are marked with a colored star to indicate the sanctuary of origin (purple = TCRC, blue = NICS). Virus names are followed by (host, location, year, GenBank accession number). Bootstrap values (%) ≥ 50 are represented by numbers beside branches (1000 replicates). Scale bar is equal to nucleotide substitutions per site. (TIF) [file pone.0288007.s002.tif]

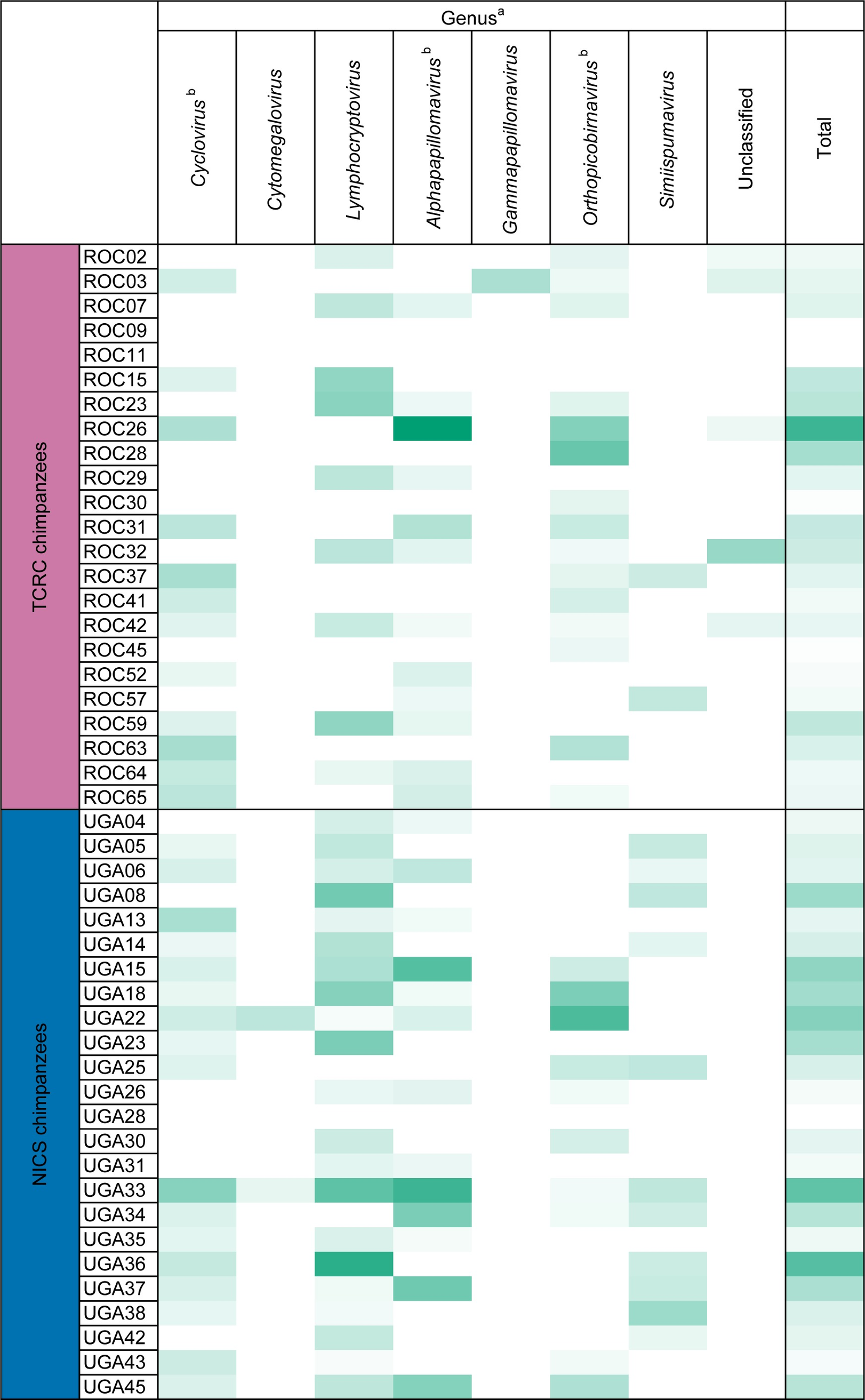

Supplement: S3 Fig — Displays viral abundance data (log10vRPM/kb) for each genus and total viral abundance data (log10vRPM/kb for all viruses) for each individual at each sanctuary. Values range from 0 (lightest) to 4.22 (darkest). a Genus refers to Table 1 and Fig 2. b For individuals infected with more than one virus from a genus, the average viral abundance is shown. (TIF) [file pone.0288007.s003.tif]
