# Supplementary material for: Viruses in saliva from sanctuary chimpanzees (Pan troglodytes) in Republic of Congo and Uganda
Source: PLoS One. 2023 Jun 29;18(6):e0288007. doi: 10.1371/journal.pone.0288007 (PMC10310015; doi:10.1371/journal.pone.0288007)
Supplement: S1 File — (DOCX) [file pone.0288007.s021.docx]

Supplementary References

1. Mugisha L, Kücherer C, Ellerbrok H, Junglen S, Opuda-Asibo J, Joseph OO, et al. Multiple Viral Infections in Confiscated Wild Born Semi-Captive Chimpanzees (*Pan troglodytes schweinfurthii*) in a Sanctuary in Uganda: Implications for Sanctuary Management and Conservation. Proc AAZV Conf. 2011;190–5.
2. Wevers D, Metzger S, Babweteera F, Bieberbach M, Boesch C, Cameron K, et al. Novel Adenoviruses in Wild Primates: A High Level of Genetic Diversity and Evidence of Zoonotic Transmissions. J Virol. 2011 Oct 15;85(20):10774–84.
3. Dunay E, Owens LA, Dunn CD, Rukundo J, Atencia R, Cole MF, et al. Viruses in sanctuary chimpanzees across Africa. Am J Primatol. 2023 Jan;85(1):e23452.
4. Thom K, Morrison C, Lewis JCM, Simmonds P. Distribution of TT virus (TTV), TTV-like minivirus, and related viruses in humans and nonhuman primates. Virology. 2003 Feb 15;306(2):324–33.
5. Mombo IM, Berthet N, Lukashev AN, Bleicker T, Brünink S, Léger L, et al. First Detection of an Enterovirus C99 in a Captive Chimpanzee with Acute Flaccid Paralysis, from the Tchimpounga Chimpanzee Rehabilitation Center, Republic of Congo. PLoS ONE. 2015 Aug 24;10(8):e0136700.
6. Lyons S, Sharp C, LeBreton M, Djoko CF, Kiyang JA, Lankester F, et al. Species Association of Hepatitis B Virus (HBV) in Non-Human Apes; Evidence for Recombination between Gorilla and Chimpanzee Variants. PLoS One. 2012 Mar 14;7(3):e33430.
7. MacDonald DM, Holmes EC, Lewis JCM, Simmonds P. Detection of Hepatitis B Virus Infection in Wild-Born Chimpanzees (*Pan troglodytes verus*): Phylogenetic Relationships with Human and Other Primate Genotypes. J Virol. 2000 May;74(9):4253–7.
8. Makuwa M, Souquière S, Telfer P, Leroy E, Bourry O, Rouquet P, et al. Occurrence of hepatitis viruses in wild-born non-human primates: a 3 year (1998-2001) epidemiological survey in Gabon. J Med Primatol. 2003 Dec;32(6):307–14.
9. Mugisha L, Kaiser M, Ellerbrok H, Pauli G, Opuda-Asibo J, Joseph OO, et al. The “original” Hepatitis B virus of Eastern chimpanzees (*Pan trogrodytes schweinfurthii*). Virus Res. 2011 Jan 1;155(1):372–5.
10. Njouom R, Mba SAS, Nerrienet E, Foupouapouognigni Y, Rousset D. Detection and characterization of hepatitis B virus strains from wild-caught gorillas and chimpanzees in Cameroon, Central Africa. Infect Genet Evol. 2010 Aug;10(6):790–6.
11. Starkman SE, MacDonald DM, Lewis JCM, Holmes EC, Simmonds P. Geographic and species association of hepatitis B virus genotypes in non-human primates. Virology. 2003 Sep 15;314(1):381–93.
12. Mugisha L, Leendertz FH, Opuda-Asibo J, Olobo JO, Ehlers B. A novel herpesvirus in the sanctuary chimpanzees on Ngamba Island in Uganda. J Med Primatol. 2010b;39(1):71–6.. Harvala H, McIntyre CL, Imai N, Clasper L, Djoko CF, LeBreton M, et al. High Seroprevalence of Enterovirus Infections in Apes and Old World Monkeys. Emerg Infect Dis. 2012 Feb;18(2):283–6.
13. Mombo IM, Boundenga L, Berthet N, Atencia R, Cox D, Maganga GD, et al. A Second Genome Sequence of an Enterovirus C99 Detected in a Healthy Chimpanzee. Microbiol Resour Announc. 2020;9(42):e00893-20.
14. Sadeuh-Mba SA, Bessaud M, Joffret ML, Zanga MCE, Balanant J, Ngole EM, et al. Characterization of Enteroviruses from Non-Human Primates in Cameroon Revealed Virus Types Widespread in Humans along with Candidate New Types and Species. PLoS Negl Trop Dis. 2014 Jul 31;8(7):e3052.
15. Scuda N, Madinda NF, Akoua-Koffi C, Adjogoua EV, Wevers D, Hofmann J, et al. Novel Polyomaviruses of Nonhuman Primates: Genetic and Serological Predictors for the Existence of Multiple Unknown Polyomaviruses within the Human Population. PLoS Pathog. 2013 Jun 20;9(6):e1003429.
16. Calattini S, Betsem EBA, Froment A, Mauclère P, Tortevoye P, Schmitt C, et al. Simian Foamy Virus Transmission from Apes to Humans, Rural Cameroon. Emerg Infect Dis. 2007 Sep;13(9):1314–20.
17. Mugisha L, Kucherer C, Ellerbrok H, Junglen S, Opuda-Asibo J, Joseph OO, et al. Retroviruses in Wild-Born Semi-Captive East African Sanctuary Chimpanzees (*Pan troglodytes schweinfurthii*). Open Vet Sci J. 2010a;4(1):6–10.
